# Sex differences of neutrophil to high-density lipoprotein cholesterol ratio in predicting the severity of coronary lesions in acute coronary syndrome patients

**DOI:** 10.1186/s12944-025-02478-w

**Published:** 2025-02-17

**Authors:** Chuntian Wang, Kun Shang, Lina Cao, Jiangying Kuang, Xiang Ning, Huiqiang Chen

**Affiliations:** 1 Department of Pediatric Surgery, The Second Hospital, Shandong University, Jinan, Shandong China; 2 Department of Pediatric Oncology, Shandong Cancer Hospital and Institute, Shandong First Medical University, Shandong Academy of Medical Sciences, Jinan, Shandong China; 3 Department of Cardiology, The Second Hospital, Shandong University, Jinan, Shandong China

**Keywords:** Acute coronary syndrome, Sex characteristics, Neutrophils, High density lipoprotein cholesterol, Coronary stenosis

## Abstract

**Background:**

The neutrophil-to-high-density lipoprotein cholesterol ratio (NHR) is closely related to the occurrence and severeness of coronary stenosis in subjects with acute coronary syndrome (ACS). Men have higher NHR levels than women. The study was designed to examine the usefulness of NHR for predicting severe coronary stenosis in male and female ACS subjects.

**Methods:**

We enrolled 165 ACS patients (105 males and 60 females) who underwent coronary angiography. Clinical parameters; fasting glucose, creatinine, and uric acid levels; lipid profiles; and routine blood indices were measured. The NHR was computed by dividing the neutrophil numbers by the concentration of high-density lipoprotein cholesterol. Coronary stenosis severity was assessed using the Gensini score (GS). A GS˃41 points was regarded as a high GS.

**Results:**

The NHR was clearly greater in male ACS individuals than in their female counterparts (*P* = 0.001). The NHR was positively linked to the GS (*r* = 0.330, *P* = 0.001) in men, whereas there was no association between NHR and the GS in women (*r*=-0.0.032, *P* = 0.805). Univariate logistic regression analyses indicated that the NHR predicted a high GS in male ACS patients but not in their female counterparts. Multivariate logistic regression analyses indicated that a high NHR was an independent marker of severe coronary lesions in men (*P* = 0.001) but not in women (*P* = 0.274). The areas under the receiver operating characteristic curve of the NHR for the prediction of severe atherosclerosis were 0.681 (*P* = 0.001) and 0.560 (*P* = 0.431) in male and female subjects, respectively. An NHR value > 3.99 had a specificity and a sensitivity of 68% and 64%, respectively, for forecasting severe atherosclerosis in male subjects.

**Conclusions:**

The NHR could be utilised to independently predict the severeness of coronary lesions in male ACS subjects but not in their female counterparts. Therefore, the NHR should be used with caution when screening female ACS patients for severe coronary restrictions.

**Supplementary Information:**

The online version contains supplementary material available at 10.1186/s12944-025-02478-w.

## Background

Acute coronary syndrome (ACS), one serious condition of cardiovascular disease, needs urgent evaluation and management. Part of the causes of atherosclerosis and ACS are inflammation and dyslipidemia [1, 2]. It is commonly accepted that neutrophils, as proinflammatory cells, play active parts in the origination, disease development, and aggravation of atherosclerosis [3, 4]. High-density lipoprotein cholesterol (HDL-C), in contrast, not only facilitates cholesterol flowing out of the arterial wall but also can effectively combat atherosclerosis and inflammation [5]. Thus, the dynamically changing of neutrophils and HDL-C might strongly influence the origination and progression of atherosclerosis and ACS.

An integrated parameter, the neutrophil-to-HDL-C ratio (NHR) was introduced to mimic both inflammation and dyslipidemia recently. Investigations revealed that the NHR could be used as a short-term prognostic index in subjects who received coronary interventional therapy due to ST-segment elevation myocardial infarction (STEMI) [6, 7]. One study recently demonstrated that high NHR levels means greater risk of recurrent myocardial infarction and death in aged subjects suffering from acute myocardial infarction [8]. In addition, Ozgeyik and colleagues reported that the NHR offered better mortality prediction than other parameters did after total occlusion of the coronary arteries [9]. Among subjects with coronary heart disease, poor prognosis is associated with severe coronary lesions. Several studies have revealed that the NHR is not only linked to the origination and severeness of coronary lesions but is also an effective marker for assessing coronary stenosis severity in individuals suffering from non-ACS [10, 11] or ACS [6, 12]. Notably, men have lower HDL-C concentration [13–15] and higher neutrophil and NHR levels [16, 17] than women do. However, to the best of our knowledge, it has not been determined whether sex-related alterations in the NHR can predict the severe coronary stenosis in subjects with ACS. Hence, the aim of current research was to examine the value of NHR for forecasting severe coronary stenosis in male and female ACS patients. We hypothesized that there is a sex difference in the usefulness of the NHR for predicting the severeness of coronary atherosclerotic lesions.

## Methods

### Patients

The research was conducted using a cross-sectional research design. The subjects were selected from hospitalized patients who were subjected to diagnostic coronary angiography due to chest tightness or pain in our centre between September 2020 and December 2020. The screening criterion was ACS status [18]. The exclusion criteria were heart failure, coronary artery spasm, coronary artery bypass surgery, systemic inflammatory disease, local or systemic infection, haematological diseases, rheumatic diseases, malignant neoplasms, renal dysfunction and hepatic insufficiency. 165 ACS patients (men = 105; women = 60) were ultimately enrolled in this study. There were 13 subjects with STEMI, including 10 men and 3 women. The research proposal was endorsed by the board of institutional ethics. The study was done in accordance with the guiding principles of the Declaration of Helsinki.

### Demographic and anthropometric measurements

Data were collected for the following participant characteristics: age; sex; systolic blood pressure (SBP); diastolic blood pressure (DBP); body mass index (BMI) and history of smoking, hypertension and diabetes. The calculation of BMI was performed by dividing the weight in kilograms by the height in meters squared. Current use of antihypertensive drugs, SBP ≥ 140 mmHg or DBP ≥ 90 mmHg were considered indicators of hypertension. Diabetes was diagnosed according to the use of oral hypoglycaemic agents or insulin [19].

### Laboratory analysis

The analyses were conducted in hospital laboratories that are accredited by the relevant authorities. Blood samples were taken after a fast for 12 h from dinner to the next morning. The serum platelet numbers, white blood cell (WBC) numbers, neutrophil numbers, and haemoglobin levels were conducted utilising an automatic analyser. The concentrations of HDL-C, creatinine, fasting glucose, uric acid (UA), total cholesterol (TC), total triglyceride (TG) and low-density lipoprotein cholesterol (LDL-C) was conducted by a standard protocol on an automatic biochemistry analyser. The NHR was computed by dividing the neutrophil numbers by the HDL-C concentration.

### Angiographic analysis

Selective coronary angiography, which was scheduled for the same day or the next day after the blood test, was conducted using the radial artery approach technique. Coronary angiograms were blindly reviewed and evaluated by two cardiologists who were not privy to the specific patient information. As previously reported [20], the severeness of coronary stenosis can be assessed with the Gensini scoring system. The Gensini score (GS) assigned to each case of coronary atherosclerosis lesions was determined on the basis of geographic relevance and severity degree of the luminal lesions [20, 21]. The male and female participants were divided into two subgroups, i.e., the low/intermediate-GS group (GS ≤ 41) and the high-GS group (GS˃41) [20], by the Gensini scores.

### Statistical analyses

The distribution pattern was determined using the Shapiro-Wilk test. The means ± standard deviations and independent samples were used to depict the continuous variables with a Gaussian distribution. Student’s t-tests was performed to compare the means of the groups. The Mann-Whitney U test was used to express continuous variables having a non-Gaussian distribution as medians with 25th and 75th percentiles. The chi-square test was carried out to compare categorical variables, which are represented as frequency percentages. Pearson’s correlation coefficient was conducted to do correlation analysis. To ascertain the relationship between the NHR and a GS > 41 points, logistic regression analysis were utilised. For male and female ACS patients, respectively, variables that showed a *P*-value of less than 0.05 or 0.1 in the univariate analyses were then included in the multivariate analyses. In order to determine appropriate NHR cut-off levels for forecasting the severity of coronary lesions, receiver operating characteristic (ROC) curve analysis was employed. The analysis of the data was conducted utilising the IBM SPSS software package for Windows 23.0. *P* < 0.05 was considered to be significance.

## Results

165 subjects with ACS (105 males and 60 females, mean age: 62.7 ± 11.0 years) were included in this study. Compared with female ACS subjects, male ACS patients presented significantly higher NHRs (4.52 ± 2.34 vs. 3.40 ± 1.55, *P* = 0.001). According to their GS, the male or female subjects were split up into two groups: the high-GS group (GS > 41 points) and the low/intermediate-GS group (GS 1 to 41 points).

As shown in Table 1, the characteristics of the enrolled patients classified by sex and coronary lesions severity are summarized. Male patients in the high-GS group were more senior than their counterparts in the low/intermediate-GS group (*P* = 0.003). In addition, compared with the controls, male subjects in the high-GS group presented lower haemoglobin (*P* = 0.011) and higher NHRs (*P* < 0.001), leukocyte counts (*P* < 0.001) and neutrophil counts (*P* < 0.001). Nevertheless, no notable distinction was found between the two male groups with respect to SBP, DBP, BMI, smoking status, diabetes status, hypertension status, fasting glucose, platelets, creatinine, UA or lipid profile. As indicated in Table 1, female subjects in the high-GS group seemed to be older than their counterparts in the low/intermediate-GS group (*P* = 0.056). Nevertheless, no notable distinction was found between the two female groups concerning the remaining demographic, biochemical and haematological parameters.

**Table 1:** Baseline characteristics of the study population

Variables		Male			Female	
	low/intermediate-GS group (≤ 41, *N* = 53)	high-GS group (>41, *N* = 52)	*P* value	low/intermediate-GS group (≤ 41, *N* = 25)	high-GS group (>41, *N* = 35)	*P* value
Age (year)	57.26 ± 10.23	64.04 ± 12.20	0.003	63.76 ± 8.94	68.03 ± 7.91	0.056
BMI (kg/m^2^)	26.48 ± 3.27	25.49 ± 2.85	0.102	25.12 ± 4.57	25.51 ± 3.17	0.696
SBP (mmHg)	138.55 ± 16.83	141.73 ± 21.27	0.396	142.60 ± 18.44	142.54 ± 18.12	0.991
DBP (mmHg)	87.11 ± 10.10	84.87 ± 11.26	0.284	84.20 ± 13.24	84.09 ± 10.78	0.971
Smoking (%)	29(54.7)	26(50.0)	0.628	1(4.0)	4(11.4)	0.580
Hypertension (%)	38(71.7)	41(78.8)	0.396	14(56.0)	23(65.7)	0.445
Diabetes (%)	12(22.6)	13(25.0)	0.777	8(32.0)	14(40.0)	0.526
Fasting glucose (mmol/L)	5.99 ± 2.01	5.98 ± 1.89	0.975	5.90 ± 1.50	6.20 ± 1.92	0.516
TC (mmol/L)	3.90 ± 0.95	4.18 ± 1.04	0.147	4.35 ± 1.14	4.93 ± 1.36	0.087
LDL-C (mmol/L)	2.25 ± 0.82	2.50 ± 0.90	0.146	2.46 ± 0.87	2.98 ± 1.19	0.068
HDL-C (mmol/L)	1.06 ± 0.25	1.00 ± 0.22	0.175	1.15 ± 0.30	1.14 ± 0.25	0.821
TG (mmol/L)	1.06(0.72–1.57)	1.30(0.88–1.81)	0.548	1.18(0.85–1.55)	1.47(1.10–1.94)	0.743
Haemoglobin (g/L)	148.40 ± 13.91	140.96 ± 15.55	0.011	127.68 ± 10.21	126.11 ± 13.78	0.632
Platelets (10^9^/L)	227.21 ± 71.76	218.40 ± 50.56	0.470	201.72 ± 54.52	226.00 ± 47.25	0.071
WBC (10^9^/L)	6.28 ± 1.13	7.63 ± 2.33	0.000	5.89 ± 1.31	6.58 ± 2.05	0.134
Neutrophil (10^9^/L)	3.74 ± 1.03	4.98 ± 1.96	0.000	3.50 ± 1.19	3.88 ± 1.67	0.338
NHR	3.73 ± 1.42	5.33 ± 2.80	0.000	3.24 ± 1.48	3.52 ± 1.61	0.482
Creatinine (µmol/L)	77.36 ± 11.15	77.78 ± 13.93	0.863	59.92 ± 10.45	62.57 ± 13.47	0.414
UA (µmol/L)	331.72 ± 98.95	332.53 ± 92.28	0.966	243.88 ± 77.46	280.22 ± 109.48	0.160
GS	15.96 ± 12.08	90.23 ± 41.85	0.000	13.06 ± 12.11	90.90 ± 39.84	0.000

BMI body mass index, SBP systolic blood pressure, DBP diastolic blood pressure, TC total cholesterol, LDL-C low-density lipoprotein cholesterol, HDL-C high-density lipoprotein cholesterol, TG triglyceride, WBC white blood cell, NHR neutrophils to HDL-C ratio, UA uric acid, GS Gensini score

To explore the linkage between the NHR and the GS in ACS patients, pearson correlation analysis was employed. The NHR was connected to the GS (*r* = 0.203, *P* = 0.009) in all ACS subjects. The NHR was strongly linked to the GS (*r* = 0.330, *P* = 0.001) in male ACS participants according to the data in Table 2. Nevertheless, no discernible connection was detected between the NHR and the GS in female patients (*r*=-0.0.032, *P* = 0.805).

**Table 2:** Correlation coefficients of NHR with gensini score

		Gensini score			
	Male			Female	
Parameters	Correlation coefficients		*P* value	Correlation coefficients	*P* value
NHR	0.330		0.001	-0.032	0.805

NHR neutrophils to high-density lipoprotein cholesterol ratio

To further examine the independent predictors for severe obstructions in the coronary arteries, analyses of univariate and multivariate regression were conducted.

Univariate analyses indicated that age, haemoglobin and the NHR predicted a high GS (> 41 points) in male ACS patients, as shown in Table 3. After multivariate analysis, a high NHR was independently correlated to severe coronary atherosclerosis (OR: 1.558, 95% CI = 1.202–2.020, *P* = 0.001), as was age (OR: 1.054, 95% CI = 1.009–1.101, *P* = 0.018).

**Table 3:** Logistic regression analyses to identify independent predictors of a high gensini score in male patients

Variables	Univariate OR (95% CI)	*P* value	Multivariate OR (95% CI)	*P* value
Age	1.055(1.017–1.095)	0.004	1.054(1.009–1.101)	0.018
Haemoglobin	0.966(0.940–0.993)	0.014	0.979(0.949–1.011)	0.191
NHR	1.486(1.173–1.882)	0.001	1.558(1.202–2.020)	0.001

OR odds ratio, CI confidence interval, NHR neutrophils to high-density lipoprotein cholesterol ratio

As demonstrated in Table 4, platelet count (OR: 1.017, 95% CI = 1.002–1.032, *P* = 0.024) and age (OR: 1.126, 95% CI = 1.033–1.229, *P* = 0.007) were correlated to severe coronary atherosclerosis in female subjects. However, the NHR (OR: 1.273, 95% CI = 0.826–1.962, *P* = 0.274) was not an indicator of severe coronary lesions in female subjects with ACS.

**Table 4:** Logistic regression analyses to identify independent predictors of a high gensini score in female patients

Variables	Univariate OR (95% CI)	*P* value	Multivariate OR (95% CI)	*P* value
Age	1.065(0.997–1.138)	0.063	1.126(1.033–1.229)	0.007
TC	1.465(0.939–2.286)	0.092	1.568(0.465–5.287)	0.469
LDL-C	1.641(0.951–2.834)	0.075	0.994(0.236–4.190)	0.994
Platelets	1.010(0.999–1.021)	0.076	1.017(1.002–1.032)	0.024
NHR	1.136(0.799–1.617)	0.478	1.273(0.826–1.962)	0.274

OR odds ratio, CI confidence interval, TC total cholesterol, LDL-C low-density lipoprotein cholesterol, NHR neutrophils to high-density lipoprotein cholesterol ratio

**Fig. 1:**
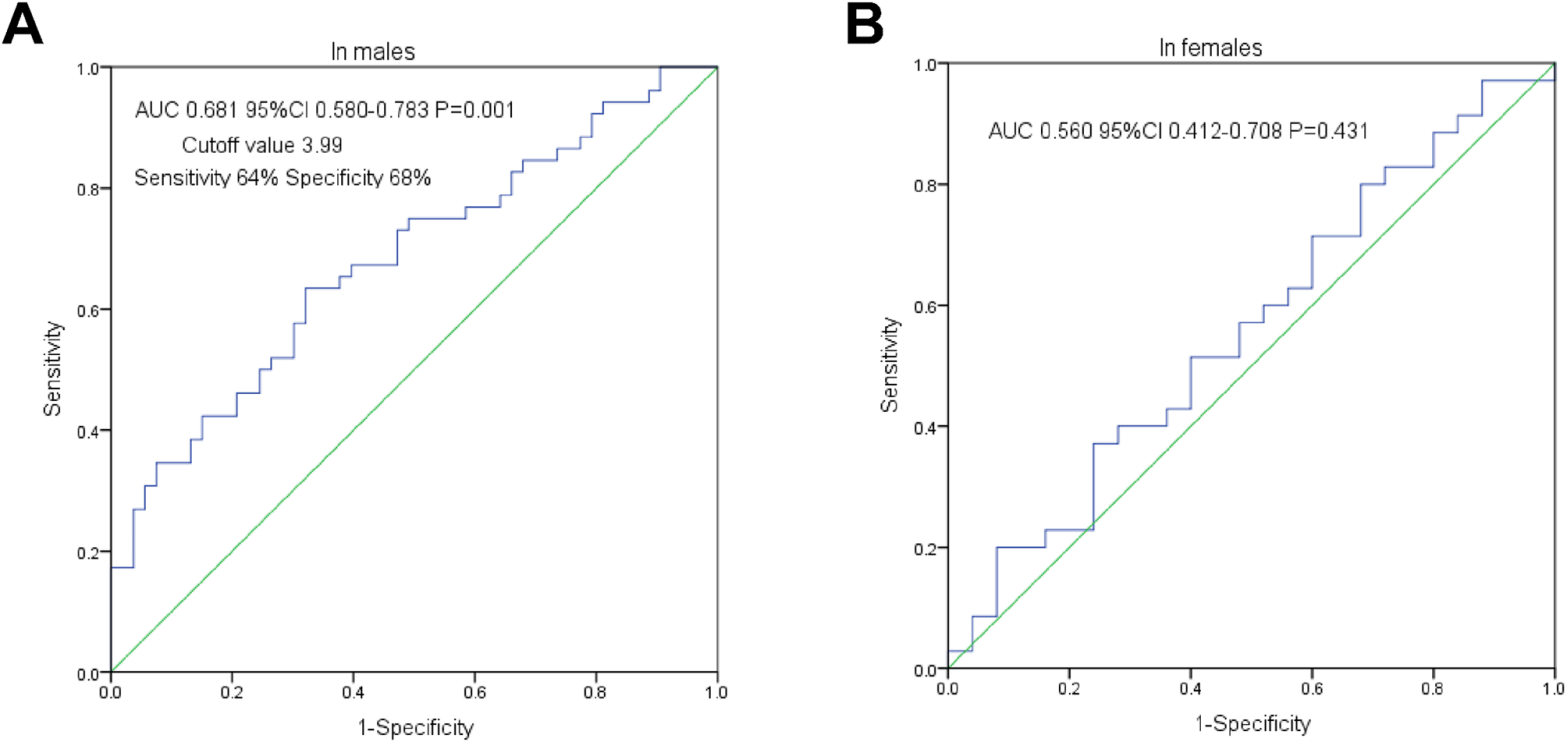
ROC curve analysis of NHR for predicting high gensini score in male (**A**) or in female (**B**) ACS patients. The AUC analysis revealed that NHR was a significant predictor of a high gensini score in males (**A**) but not in females (**B**). The NHR cutoff value of 3.99 predicts a high gensini score with a specificity of 68.0% and sensitivity of 64% in male ACS subjects. ROC receiver operating characteristic, ACS acute coronary syndrome, AUC area under curve, CI confidence interval, NHR neutrophils to high-density lipoprotein cholesterol ratio

ROC curve analysis of the NHR for forecasting severe coronary atherosclerosis (> 41 points) in male or female ACS participants are displayed in Fig. 1. As shown in Fig. 1A and B, the area under the ROC curve (AUC) of the NHR for the indication of severe atherosclerosis was 0.681 (95% CI: 0.580–0.783, *P* = 0.001) in male subjects, whereas the NHR was not an indicator of severe atherosclerosis in female subjects (AUC = 0.560, 95% CI: 0.412–0.708, *P* = 0.431). Additionally, an NHR value > 3.99 had a specificity and a sensitivity of 68% and 64%, respectively, for forecasting severe coronary atherosclerosis in male patients.

## Discussion

The study focused on sex-related differences in the usefulness of NHR for forecasting the severeness of coronary lesions in ACS patients. The NHR was not only positively associated with the severeness of coronary atherosclerosis lesions but also independently predicted severe atherosclerosis in male ACS patients but not in their female counterparts.

As mentioned in the Introduction section, neutrophils play active parts in the occurrence and progression of atherosclerosis and ACS [3, 4], whereas HDL-C has potent anti-atherosclerosis and anti-inflammatory properties [5]. In this context, the NHR, as an integrated index, is used to reflect both inflammation and lipid metabolism. Here, we discovered that compared with female subjects, male ACS patients had reduced amounts of HDL-C and greater quantities of NHR and neutrophil. Our data are in line with those of previous reports [13–17]. Although the mechanisms for sex-associated alterations in the NHR are not fully understood, the neutrophil numbers and HDL-C levels could be influenced by many factors, including sex and age. Women under age 50 had higher neutrophil levels than women aged 51–70 years old while neutrophil levels were shown to increase gradually with age in men [17]. Cheung et al. reported that the decrease in the serum HDL-C concentration with age in Chinese females contrasted with that reported in American females, whereas the serum HDL-C concentration did not markedly change with age in men [14]. This finding was supported by later studies, which demonstrated that serum HDL-C was greater in premenopausal women than in postmenopausal women [22]. However, as reported in a recent study, multivariable-adjusted estimated annual changes in HDL-C increased observably in female subjects after the age of 60 [13]. To complicate matters, genetically low HDL-C is relevant to high peripheral white blood cells, as shown in a Mendelian randomization study [23]. On the basis of these findings, the sex-related differences in the NHR could be attributed in part to the dramatic changes in neutrophil and HDL-C levels with age in women.

In recent years, growing research has suggested that the NHR could be used for risk identification in subjects with stable ischaemic heart disease as well as those with ACS [2, 6, 10–12, 24]. For example, studies have revealed that the NHR not only exhibits a positive correlation with coronary lesions but also is a good indicator of severe coronary stenosis in subjects diagnosed with stable coronary lesions [10, 11]. Similarly, Ren et al. [12] and Guo et al. [6] both uncovered that the NHR has a close relationship with coronary artery lesions and could independently forecast the severeness of coronary lesions in ACS individuals. Further research by Liu and colleagues showed that the NHR and the severeness of coronary atherosclerotic stenosis were still connected although LDL-C was well controlled [24]. However, Manoochehri et al. reported that the NHR had no significant correlation with the GS, although the NHR was considered an independent variable for the prediction of the GS in the regression model [16]. A possible explanation for this might be the small sample size in the above study. Consistent with the previous reports above, the present investigation demonstrated that the NHR has positive association with the GS in male subjects with ACS, whereas no obvious correlation was found between the NHR and the GS in female participants. Logistic regression analyses showed that NHR was an independent predictive marker of severe coronary lesions in male ACS patients but not in female patients. We also discovered that the NHR is not effective in screening female patients with severe coronary artery stenosis. Thus, our study supported the view that the NHR could be used as a valid parameter for identifying severe coronary atherosclerotic lesions in male ACS patients but not in female patients. Moreover, several studies revealed that the NHR could serve as a short-term or long-term prognostic marker in patients with ACS [2, 6–9]. To the best of our knowledge, whether the prognostic value of the NHR is the same for men and women has not been clarified. Considerably more work will need to be done to determine gender-associated alterations in the NHR for indicative of mortality in ACS individuals.

There are sex-related discrepancies in the etiology, the pathogenesis and the diagnosis of coronary atherosclerotic lesions [25]. The most unexpected finding of our study was that the NHR was not an effective marker for indicative of severe coronary atherosclerotic lesions in women. Although we are not certain, one possible explanation might be that the course of atherosclerosis and ACS in women is unique, shorter than that in men, and cannot be reflected properly by the parameters reflecting inflammation and lipid metabolism, as in men. In general, females initially present with coronary atherosclerotic heart disease almost 10 years later than males do [26]. The incidence of coronary atherosclerotic disease among women exhibits a marked increase following the onset of menopause, whereas coronary atherosclerotic disease has a relatively low incidence in women before menopause [26–29]. The above increase could be attributed mainly to the loss of oestrogen and its cardioprotective effects in postmenopausal women, considering that oestrogen could increase arterial vasodilatation, suppress the response of blood vessels to injury and prevent atherosclerosis [26–29]. In addition, previous studies have shown that oestradiol increases neutrophil survival in women [30] and that estrone is related to serum HDL-C levels in older women [31], indicating that the NHR might be influenced by oestrogen in older women. In addition to hormones, however, the discrepancies between males and females in patterns of cardiovascular disease could be attributed to differences in cardiovascular risk factors [32]. The sex-related distinctions between BMI and body fat distribution and metabolic phenotypes may significantly influence coronary plaque composition and clinical prognosis [33]. In addition to traditional risk factors, females have sex-related cardiovascular risk factors, including gestational diabetes mellitus, preeclampsia, preterm delivery and polycystic ovary syndrome [22]. Women are predisposed to undiagnosed cardiovascular risk factors, such as physical abuse, autoimmune disorders, anxiety or depression, and cardiotoxic breast cancer treatment [22]. These factors might simultaneously affect the NHR and the coronary lesions, contributing to the inconsistency of the NHR and the severeness of coronary lesions. Further research is necessary to determine the correlation of NHR with the risk of developing coronary atherosclerotic stenosis in female ACS individuals.

Consistent with previous reports [10, 34], the present study indicated that age was an independent predictive marker of severe coronary artery lesions in male and female ACS patients. A previous study revealed that men had higher levels of haemoglobin and white blood cells than their women did [15], which was consistent with our findings. White blood cells were markedly increased in male subjects with coronary artery disease, whereas no significant changes were detected in female subjects with coronary artery disease [34], which was consistent with our findings. Haemoglobin and platelet levels were found to be predictive factors for severe coronary artery stenosis in ACS patients in males and females, respectively, in this study, which was not supported by a previous study [15]. However, other traditional cardiovascular risk factors, including hypertension status, diabetes status, LDL-C levels, and TC levels were not independently liked to the GS in male or female ACS patients. The divergent outcomes above mentioned may be attributable to the heterogeneity of the inclusion criteria and the composition of the samples.

The NHR is a cheap and simple laboratory marker. Recent research has indicated that the NHR possesses superior diagnostic value compared to the neutrophil-to-lymphocyte ratio(NLR), while it is comparable to the highly sensitive C-reactive protein related parameter in the prediction of coronary atherosclerotic lesions [35]. Gao et al. also discovered that the performance of the NHR was better than that of neutrophil or lipid parameters in screening for severe coronary arteriosclerosis stenosis in subjects exhibiting stable coronary heart disease [11]. Furthermore, Ozgeyik and colleagues reported that the NHR offered better mortality prediction than other parameters did after total occlusion of the coronary arteries [9]. In addition, the combined NLR and LDL-C/HDL-C ratio is better than the single parameter in predicting severe coronary stenosis in ACS subjects [36]. Consequently, the NHR, coupled with other clinical parameters, is expected to be an effective biomarker for the early recognition and risk stratification of ACS, especially in males.

### Study strengths and limitations

It was a real-world study with little human intervention, and the results are relatively reliable. However, the present study is subject to a number of limitations. First, the present study population was modest in size, which may have reduced its statistical effectiveness. Second, all the subjects enrolled in the current study underwent coronary angiography, which could not be extrapolated to the general population. Third, long-term tracking data are lacking due to the cross-sectional feature of this study.

## Conclusions

In summary, the NHR was independently associated with severe coronary lesions in male ACS patients but not in their female counterparts. Thus, the NHR should be used with caution when screening ACS subjects with severe coronary artery restrictions in women. For male subjects with ACS, it is necessary to measure the NHR early to better diagnose, treat and care for these patients.

## Electronic supplementary material

Below is the link to the electronic supplementary material.

Supplementary Material 1

Supplementary Material 2

## Data Availability

No datasets were generated or analysed during the current study.

## Abbreviations

ACS: Acute coronary syndrome
BMI: Body mass index
DBP: Diastolic blood pressure
GS: Gensini score
HDL-C: High-density lipoprotein cholesterol
LDL-C: Low-density lipoprotein cholesterol
NHR: Neutrophil to high-density lipoprotein cholesterol ratio
ROC: Receiver operating characteristic
SBP: Systolic blood pressure
STEMI: ST-segment elevation myocardial infarction
TC: Total cholesterol
TG: Total triglyceride
UA: Uric acid
WBC: White blood cell
