# Supplementary material for: Sex differences of neutrophil to high-density lipoprotein cholesterol ratio in predicting the severity of coronary lesions in acute coronary syndrome patients
Source: Lipids Health Dis. 2025 Feb 17;24:54. doi: 10.1186/s12944-025-02478-w (PMC11831777; doi:10.1186/s12944-025-02478-w)
Supplement: Supplementary file 1 — Supplementary Material 1 [file 12944_2025_2478_MOESM1_ESM.pdf]

SPRINGER NATURE
Author Services

Editing Certificate

This document certifies that the manuscript

Sex differences of neutrophil to high-density lipoprotein cholesterol ratio in predicting the severity of coronary lesions in acute coronary syndrome patients

prepared by the authors

Chuntian Wang, Kun Shang, Lina Cao, Jiangying Kuang, Xiang Ning, Huiqiang Chen

was edited for proper English language, grammar, punctuation, spelling, and overall style by one or more of the highly qualified English speaking editors at SNAS.

This certificate was issued on **January 19, 2025** and may be verified on the SNAS website using the verification code **C687-04B2-1FCB-7FA7-7E82**.

Neither the research content nor the authors' intentions were altered in any way during the editing process. Documents receiving this certification should be English-ready for publication; however, the author has the ability to accept or reject our suggestions and changes. To verify the final SNAS edited version, please visit our verification page at secure.authorservices.springernature.com/certificate/verify.
If you have any questions or concerns about this edited document, please contact SNAS at support@as.springernature.com.

SNAS provides a range of editing, translation, and manuscript services for researchers and publishers around the world.
For more information about our company, services, and partner discounts, please visit authorservices.springernature.com.
