# Supplementary material for: Sex differences of neutrophil to high-density lipoprotein cholesterol ratio in predicting the severity of coronary lesions in acute coronary syndrome patients
Source: Lipids Health Dis. 2025 Feb 17;24:54. doi: 10.1186/s12944-025-02478-w (PMC11831777; doi:10.1186/s12944-025-02478-w)
Supplement: Supplementary file 2 — Supplementary Material 2 [file 12944_2025_2478_MOESM2_ESM.pdf]

Sex differences of neutrophil to high density lipoprotein cholesterol ratio in predicting the severi

MRE Press

Document Details

Submission ID

trn:oid:::9177:80206167

Submission Date

Jan 26, 2025, 11:11 PM GMT+8

Download Date

Jan 26, 2025, 11:12 PM GMT+8

File Name

2579440204827418620941_Sex differences of neutrophil to high density lipoprotein cholesterol ….docx

File Size

27.5 KB

9 Pages

4,438 Words

25,905 Characters

15% Overall Similarity

The combined total of all matches, including overlapping sources, for each database.

Filtered from the Report

- Bibliography

Match Groups

- **44** Not Cited or Quoted 13%
  Matches with neither in-text citation nor quotation marks
- **7** Missing Quotations 2%
  Matches that are still very similar to source material
- **0** Missing Citation 0%
  Matches that have quotation marks, but no in-text citation
- **0** Cited and Quoted 0%
  Matches with in-text citation present, but no quotation marks

Top Sources

12% Internet sources
13% Publications
0% Submitted works (Student Papers)

Integrity Flags

0 Integrity Flags for Review

No suspicious text manipulations found.

Our system's algorithms look deeply at a document for any inconsistencies that would set it apart from a normal submission. If we notice something strange, we flag it for you to review.

A Flag is not necessarily an indicator of a problem. However, we'd recommend you focus your attention there for further review.

Match Groups

- **44** Not Cited or Quoted 13%
  Matches with neither in-text citation nor quotation marks
- **7** Missing Quotations 2%
  Matches that are still very similar to source material
- **0** Missing Citation 0%
  Matches that have quotation marks, but no in-text citation
- **0** Cited and Quoted 0%
  Matches with in-text citation present, but no quotation marks

Top Sources

12%	Internet sources
13%	Publications
0%	Submitted works (Student Papers)

Top Sources

The sources with the highest number of matches within the submission. Overlapping sources will not be displayed.

#	Type	Source	%
1	Internet	www.frontiersin.org	3%
2	Internet	www.ncbi.nlm.nih.gov	2%
3	Internet	bmccardiovascdisord.biomedcentral.com	<1%
4	Internet	assets-eu.researchsquare.com	<1%
5	Internet	www.hindawi.com	<1%
6	Internet	pubmed.ncbi.nlm.nih.gov	<1%
7	Internet	www.dovepress.com	<1%
8	Internet	www.researchgate.net	<1%
9	Publication	Iain Greer, Jeff Ginsberg, Charles Forbes. "Women's Vascular Health", CRC Press, ...	<1%
10	Internet	doi.org	<1%

11 Publication

Shicheng Yu, Haoxuan Jia, Zheng Li, Shengkai Ding et al. "Plasma levels of bacteri... <1%

12 Publication

Lei Zhang, Yusuke Takahashi. "Relationships between obsessive-compulsive disor... <1%

13 Publication

Yue Liu, Xiandu Jin, Wenjun Jia, Xiuju Guan, Hao Wu, Jiao Li, Min Cui, Hanmo Zhan... <1%

14 Internet

www.zora.uzh.ch <1%

15 Publication

Guangyong Chen, Naiping Yang, Junli Ren, Yibo He et al. "Neutrophil Counts to Hi... <1%

16 Publication

Julip Jung, Helen Hong, Hansang Lee, Sung Il Hwang, Hak Jong Lee. "Uni- and Mul... <1%

17 Publication

Ning Zhao, Jia Li, Qiu-Xia Zhang, Li Yang, Lin-Jie Zhang. "Elevated neutrophil-relat... <1%

18 Publication

Weifeng Xu, Haiwang Guan, Da Gao, Jingnan Pan, Zicheng Wang, Mahboob Alam, ... <1%

19 Internet

journals.plos.org <1%

20 Internet

cardiab.biomedcentral.com <1%

21 Internet

assets.researchsquare.com <1%

22 Internet

jcpsp.pk <1%

23 Internet

worldwidescience.org <1%

24 Internet

www.nature.com <1%

25 Internet

www.researchsquare.com <1%

---

26 Publication

Hongyun Shu, Sisi Han, Weiping Qiu, Jianhong Li, Xiaoyong Zhang, Haicui Su, Hon... <1%

---

27 Publication

YANG-JIA ZHUO, MING XI, YUE-PING WAN, WEI HUA et al. "Enhanced expression o... <1%

---

28 Internet

idus.us.es <1%

---

29 Publication

Jie Gao, Jun Lu, Wenjun Sha, Bilin Xu, Cuiping Zhang, Hongping Wang, Juan Xia, H... <1%

---

30 Publication

Richard N. Re. "Molecular Mechanisms in Hypertension", CRC Press, 2019 <1%

6 Abstract
Background: The neutrophil-to-high-density lipoprotein cholesterol ratio (NHR) is closely
related to the occurrence and severeness of coronary stenosis in subjects with acute coronary
30 syndrome (ACS). Men have higher NHR levels than women. The study was designed to
examine the usefulness of NHR for predicting severe coronary stenosis in male and female
ACS subjects.
Methods: We enrolled 165 ACS patients (105 males and 60 females) who underwent coronary
angiography. Clinical parameters; fasting glucose, creatinine, and uric acid levels; lipid profiles;
18 and routine blood indices were measured. The NHR was computed by dividing the neutrophil
numbers by the concentration of high-density lipoprotein cholesterol. Coronary stenosis
3 severity was assessed using the Gensini score (GS). A GS>41 points was regarded as a high
GS.
Results: The NHR was clearly greater in male ACS individuals than in their female counterparts
(P=0.001). The NHR was positively linked to the GS (r=0.330, P=0.001) in men , whereas there
was no association between NHR and the GS in women (r=-0.0.032, P=0.805). Univariate
logistic regression analyses indicated that the NHR predicted a high GS in male ACS patients
15 but not in their female counterparts. Multivariate logistic regression analyses indicated that a
14 high NHR was an independent marker of severe coronary lesions in men (P=0.001) but not in
2 women (P=0.274). The areas under the receiver operating characteristic curve of the NHR for
2 the prediction of severe atherosclerosis were 0.681 (P=0.001) and 0.560 (P=0.431) in male
and female subjects, respectively. An NHR value > 3.99 had a specificity and a sensitivity of
68% and 64%, respectively, for forecasting severe atherosclerosis in male subjects.
Conclusions: The NHR could be utilised to independently predict the severeness of coronary
lesions in male ACS subjects but not in their female counterparts. Therefore, the NHR should
be used with caution when screening female ACS patients for severe coronary restrictions.

Background

25 Acute coronary syndrome (ACS), one serious condition of cardiovascular disease, needs
urgent evaluation and management. Part of the causes of atherosclerosis and ACS are
inflammation and dyslipidemia [1, 2]. It is commonly accepted that neutrophils, as
proinflammatory cells, play active parts in the origination, disease development, and
21 aggravation of atherosclerosis [3, 4]. High-density lipoprotein cholesterol (HDL-C), in contrast,
not only facilitates cholesterol flowing out of the arterial wall but also can effectively combat
atherosclerosis and inflammation [5]. Thus, the dynamically changing of neutrophils and HDL-
C might strongly influence the origination and progression of atherosclerosis and ACS.

1 An integrated parameter, the neutrophil-to-HDL-C ratio (NHR) was introduced to mimic both
inflammation and dyslipidemia recently. Investigations revealed that the NHR could be used
as a short-term prognostic index in subjects who received coronary interventional therapy
8 due to ST-segment elevation myocardial infarction (STEMI) [6, 7]. One study recently
9 demonstrated that high NHR levels means greater risk of recurrent myocardial infarction and
death in aged subjects suffering from acute myocardial infarction [8]. In addition, Ozgeyik
and colleagues reported that the NHR offered better mortality prediction than other
parameters did after total occlusion of the coronary arteries [9]. Among subjects with coronary
heart disease, poor prognosis is associated with severe coronary lesions. Several studies have
revealed that the NHR is not only linked to the origination and severeness of coronary lesions

but is also an effective marker for assessing coronary stenosis severity in individuals suffering from non-ACS [10, 11] or ACS [6, 12]. Notably, men have lower HDL-C concentration [13-15] and higher neutrophil and NHR levels [16, 17] than women do. However, to the best of our knowledge, it has not been determined whether sex-related alterations in the NHR can
23 predict the severe coronary stenosis in subjects with ACS. Hence, the aim of current research was to examine the valueof NHR for forecasting severe coronary stenosis in male and female ACS patients. We hypothesized that there is a sex difference in the usefulness of the NHR for predicting the severeness of coronary atherosclerotic lesions.

Methods

Patients

The research was conducted using a cross-sectional research design. The subjects were selected from hospitalized patients who were subjected to diagnostic coronary angiography due to chest tightness or pain in our centre between September 2020 and December 2020. The screening criterion was ACS status [18]. The exclusion criteria were heart failure, coronary artery spasm, coronary artery bypass surgery, systemic inflammatory disease, local or systemic infection, haematological diseases, rheumatic diseases, malignant neoplasms, renal dysfunction and hepatic insufficiency. 165 ACS patients (men=105; women=60) were ultimately enrolled in this study. There were 13 subjects with STEMI, including 10 men and 3
10 women. The research proposal was endorsed by the board of institutional ethics. The study was done in accordance with the guiding principles of the Declaration of Helsinki.

Demographic and anthropometric measurements

7 Data were collected for the following participant characteristics: age; sex; systolic blood pressure (SBP); diastolic blood pressure (DBP); body mass index (BMI) and history of smoking,
2 hypertension and diabetes. The calculation of BMI was performed by dividing the weight in
1 kilograms by the height in meters squared. Current use of antihypertensive drugs, SBP ⩾140 mmHg or DBP ⩾90 mmHg were considered indicators of hypertension. Diabetes was diagnosed according to the use of oral hypoglycaemic agents or insulin [19].

Laboratory analysis

The analyses were conducted in hospital laboratories that are accredited by the relevant authorities. Blood samples were taken after a fast for 12 h from dinner to the next morning. The serum platelet numbers, white blood cell (WBC) numbers, neutrophil numbers, and haemoglobin level and were conducted utilising an automatic analyser. The concentrations
5 of HDL-C, creatinine, fasting glucose, uric acid (UA), total cholesterol (TC), total triglyceride (TG) and low-density lipoprotein cholesterol (LDL-C) was conducted by a standard protocol
4 on an automatic biochemistry analyser. The NHR was computed by dividing the neutrophil numbers by the HDL-C concentration.

Angiographic analysis

Selective coronary angiography, which was scheduled for the same day or the next day after the blood test, was conducted using the radial artery approach technique. Coronary angiograms were blindly reviewed and evaluated by two cardiologists who were not privy to the specific patient information. As previously reported [20], the severeness of coronary
22 stenosis can be assessed with the Gensini scoring system. The Gensini score (GS) assigned to each case of coronary atherosclerosis lesions was determined on the basis of geographic

relevance and severity degree of the luminal lesions [20, 21]. The male and female participants
16 were divided into two subgroups, i.e., the low/intermediate-GS group (GS≤41) and the high-
GS group (GS>41) [20], by the Gensini scores.

Statistical analyses

The distribution pattern was determined using the Shapiro-Wilk test. The means ± standard
deviations and independent samples were used to depict the continuous variables with a
2 Gaussian distribution. Student's t-tests was performed to compare the means of the groups.
2 The Mann-Whitney U test was used to express continuous variables having a non-Gaussian
20 distribution as medians with 25th and 75th percentiles. The chi-square test was carried out
to compare categorical variables, which are represented as frequency percentages. Pearson's
24 correlation coefficient was conducted to do correlation analysis. To ascertain the relationship
between the NHR and a GS >41 points, logistic regression analysis were utilized. For male
5 and female ACS patients, respectively, variables that showed a P-value of less than 0.05 or
0.1 in the univariate analyses were then included in the multivariate analyses. In order to
determine appropriate NHR cut-off levels for forecasting the severity of coronary lesions,
1 receiver operating characteristic (ROC) curve analysis was employed. The analysis of the data
1 was conducted utilising the IBM SPSS software package for Windows 23.0. P < 0.05 was
considered to be significance.

Results

165 subjects with ACS (105 males and 60 females, mean age: 62.7 ± 11.0 years) were
included in this study. Compared with female ACS subjects, male ACS patients presented
significantly higher NHRs (4.52±2.34 vs. 3.40±1.55, P=0.001). According to their GS, the male
1 or female subjects were split up into two groups: the high-GS group (GS >41 points) and the
low/intermediate-GS group (GS 1 to 41 points).

2 As shown in Table 1, the characteristics of the enrolled patients classified by sex and coronary
12 lesions severity are summarized. Male patients in the high-GS group were more senior than
their counterparts in the low/intermediate-GS group (P=0.003). In addition, compared with
the controls, male subjects in the high-GS group presented lower haemoglobin (P=0.011)
1 and higher NHRs (P <0.001), leukocyte counts (P < 0.001) and neutrophil counts (P <0.001).
Nevertheless, no notable distinctions was found between the two male groups with respect
to SBP, DBP, BMI, smoking status, diabetes status, hypertension status, fasting glucose,
platelets, creatinine, UA or lipid profile. As indicated in Table 1, female subjects in the high-
27 GS group seemed to be older than their counterparts in the low/intermediate-GS group
(P=0.056). Nevertheless, no notable distinction was found between the two female groups
concerning the remaining demographic, biochemical and haematological parameters.

6 To explore the linkage between the NHR and the GS in ACS patients, pearson correlation
11 analysis was employed. The NHR was connected to the GS (r=0.203, P=0.009) in all ACS
11 subjects. The NHR was strongly linked to the GS (r=0.330, P=0.001) in male ACS participants
according to the data in Table 2. Nevertheless, no discernible connection was detected
between the NHR and the GS in female patients (r=-0.0.032, P=0.805).

To further examine the independent predictors for severe obstructions in the coronary arteries,
analyses of univariate and multivariate regression were conducted.

Univariate analyses indicated that age, haemoglobin and the NHR predicted a high GS (>41
points) in male ACS patients, as shown in Table 3. After multivariate analysis, a high NHR was

1 independently correlated to severe coronary atherosclerosis (OR: 1.558, 95% CI = 1.202–2.020,
P=0.001), as was age (OR: 1.054, 95% CI = 1.009–1.101, P=0.018).

4 As demonstrated in Table 4, platelet count (OR: 1.017, 95% CI = 1.002-1.032, P=0.024) and
age (OR: 1.126, 95% CI = 1.033-1.229, P=0.007) were correlated to severe coronary
8 atherosclerosis in female subjects. However, the NHR (OR: 1.273, 95% CI = 0.826-1.962,
P=0.274) was not an indicator of severe coronary lesions in female subjects with ACS.

26 ROC curve analysis of the NHR for forecasting severe coronary atherosclerosis (>41 points)
in male or female ACS participants are displayed in Figure 1. As shown in Fig. 1A and Fig. 1B,
17 the area under the ROC curve (AUC) of the NHR for the indication of severe atherosclerosis
3 was 0.681 (95% CI: 0.580–0.783, P=0.001) in male subjects, whereas the NHR was not an
3 indicator of severe atherosclerosis in female subjects (AUC=0.560, 95% CI: 0.412–0.708,
P=0.431). Additionally, the NHR value > 3.99 had a specificity and a sensitivity of 68% and 64%,
respectively, for forecasting severe coronary atherosclerosis in male patients.

Discussion

28 The study focused on sex-related differences in the usefulness of NHR for forecasting the
13 severeness of coronary lesions in ACS patients. The NHR was not only positively associated
with the severeness of coronary atherosclerosis lesions but also independently predicted
severe atherosclerosis in male ACS patients but not in their female counterparts.

As mentioned in the Introduction section, neutrophils play active parts in the occurrence and progression of atherosclerosis and ACS [3, 4], whereas HDL-C has potent anti-atherosclerosis and anti-inflammatory properties [5]. In this context, the NHR, as an integrated index, is used to reflect both inflammation and lipid metabolism. Here, we discovered that compared with female subjects, male ACS patients had reduced amounts of HDL-C and greater quantities of NHR and neutrophil. Our data are in line with those of previous reports [13-17]. Although the mechanisms for sex-associated alterations in the NHR are not fully understood, the neutrophil numbers and HDL-C levels could be influenced by many factors, including sex and age. Women under age 50 had higher neutrophil levels than women aged 51–70 years old while neutrophil levels were shown to increase gradually with age in men [17]. Cheung et al. reported that the decrease in the serum HDL-C concentration with age in Chinese females contrasted with that reported in American females, whereas the serum HDL-C concentration did not markedly change with age in men [14]. This finding was supported by later studies, which demonstrated that serum HDL-C was greater in premenopausal women than in postmenopausal women [22]. However, as reported in a recent study, multivariable-adjusted estimated annual changes in HDL-C increased observably in female subjects after the age of 60 [13]. To complicate matters, genetically low HDL-C is relevant to high peripheral white blood cells, as shown in a Mendelian randomization study [23]. On the basis of these findings, the sex-related differences in the NHR could be attributed in part to the dramatic changes in neutrophil and HDL-C levels with age in women.

In recent years, growing research has suggested that the NHR could be used for risk identification in subjects with stable ischaemic heart disease as well as those with ACS [2, 6, 10-12, 24]. For example, studies have uncovered that the NHR not only exhibits a positive correlation with coronary lesions but also is a good indicator of severe coronary stenosis in subjects diagnosed with stable coronary lesions [10, 11]. Similarly, Ren et al. [12] and Guo et

al. [6] both uncovered that the NHR has a close relationship with coronary artery lesions and could independently forecast the severeness of coronary lesions in ACS individuals. Further research by Liu and colleagues revealed that the NHR and the severeness of coronary atherosclerotic stenosis were still connected although LDL-C was well controlled [24]. However, Manoochehri et al. reported that the NHR had no significant correlation with the GS, although the NHR was considered an independent variable for the prediction of the GS in the regression model [16]. A possible explanation for this might be the small sample size in the above study. Consistent with the previous reports above, the present investigation showed that the NHR has positive association with the GS in male subjects with ACS, whereas no obvious correlation was found between the NHR and the GS in female participants. Logistic regression analyses discovered that NHR was an independent predictive marker of severe coronary lesions in male ACS patients but not in female patients. We also discovered that the NHR is not effective in screening female patients with severe coronary artery stenosis. Thus, our study supported the view that the NHR could be used as a valid parameter for identifying severe coronary atherosclerotic lesions in male ACS patients but not in female patients. Moreover, several studies revealed that the NHR could serve as a short-term or long-term prognostic marker in patients with ACS [2, 6-9]. To the best of our knowledge, whether the prognostic value of the NHR is the same for men and women has not been clarified. Considerably more work will need to be done to determine gender-associated alterations in the NHR for indicative of mortality in ACS individuals.

There are sex-related discrepancies in the etiology, the pathogenesis and the diagnosis of coronary atherosclerotic lesions [25]. The most unexpected finding of our study was that the NHR was not an effective marker for indicative of severe coronary atherosclerotic lesions in women. Although we are not certain, one possible explanation might be that the course of atherosclerosis and ACS in women is unique, shorter than that in men, and cannot be reflected properly by the parameters reflecting inflammation and lipid metabolism, as in men. In general, females initially present with coronary atherosclerotic heart disease almost 10 years later than males do [26]. The incidence of coronary atherosclerotic disease among women exhibits a marked increase following the onset of menopause, whereas coronary atherosclerotic disease has a relatively low incidence in women before menopause [26-29]. The above increase could be attributed mainly to the loss of oestrogen and its cardioprotective effects in postmenopausal women, considering that oestrogen could increase arterial vasodilatation, suppress the response of blood vessels to injury and prevent atherosclerosis [26-29]. In addition, previous studies have shown that oestradiol increases neutrophil survival in women [30] and that estrone is related to serum HDL-C levels in older women [31], indicating that the NHR might be influenced by oestrogen in older women. In addition to hormones, however, the discrepancies between males and females in patterns of cardiovascular disease could be attributed to differences in cardiovascular risk factors [32]. The sex-related distinctions between BMI and body fat distribution and metabolic phenotypes may significantly influence coronary plaque composition and clinical prognosis [33]. In addition to traditional risk factors, females have sex-related cardiovascular risk factors, including gestational diabetes mellitus, preeclampsia, preterm delivery and polycystic ovary syndrome [22]. Women are predisposed to undiagnosed cardiovascular risk factors, such as physical abuse, autoimmune disorders, anxiety or depression, and cardiotoxic breast cancer

treatment [22]. These factors might simultaneously affect the NHR and the coronary lesions, contributing to the inconsistency of the NHR and the severeness of coronary lesions. Further research is necessary to determine the correlation of NHR with the risk of developing coronary atherosclerotic stenosis in female ACS individuals.

Consistent with previous reports [10,34], the present study indicated that age was an independent predictive marker of severe coronary artery lesions in male and female ACS patients. A previous study revealed that men had higher levels of haemoglobin and white blood cells than their women did [15], which was consistent with our findings. White blood cells were markedly increased in male subjects with coronary artery disease, whereas no significant changes were detected in female subjects with coronary artery disease [34], which was consistent with our findings. Haemoglobin and platelet levels were found to be predictive factors for severe coronary artery stenosis in ACS patients in males and females, respectively, in this study, which was not supported by a previous study [15]. However, other traditional cardiovascular risk factors, including hypertension status, diabetes status, LDL-C levels, and TC levels were not independently liked to the GS in male or female ACS patients. The divergent outcomes above mentioned may be attributable to the heterogeneity of the inclusion criteria and the composition of the samples.

The NHR is a cheap and simple laboratory marker. Recent research has indicated that the NHR possesses superior diagnostic value compared to the neutrophil-to-lymphocyte ratio, while it is comparable to the highly sensitive C-reactive protein related parameter in the prediction of coronary atherosclerotic lesions [35]. Gao et al. also discovered that the performance of the NHR was better than that of neutrophil or lipid parameters in screening for severe coronary arteriosclerosis stenosis in subjects exhibiting stable coronary heart disease [11]. Furthermore, Ozgeyik and colleagues reported that the NHR offered better mortality prediction than other parameters did after total occlusion of the coronary arteries [9]. In addition, the combined NLR and LDL-C/HDL-C ratio is better than the single parameter in predicting severe coronary stenosis in ACS subjects [36]. Consequently, the NHR, coupled with other clinical parameters, is expected to be an effective biomarker for the early recognition and risk stratification of ACS, especially in males.

Study strengths and limitations

It was a real-world study with little human intervention, and the results are relatively reliable. However, the present study is subject to a number of limitations. First, the present study population was modest in size, which may have reduced its statistical effectiveness. Second, all the subjects enrolled in the current study underwent coronary angiography, which could not be extrapolated to the general population. Third, long-term tracking data are lacking due to the cross-sectional feature of this study.

Conclusions

In summary, the NHR was independently associated with severe coronary lesions. in male ACS patients but not in their female counterparts. Thus, the NHR should be used with caution when screening ACS subjects with severe coronary artery restrictions in women. For male subjects with ACS, it is necessary to measure the NHR early to better diagnose, treat and care for these patients.

References

1. Shumilah AM, Othman AM and Al-Madhagi AK. Accuracy of neutrophil to lymphocyte

and monocyte to lymphocyte ratios as new inflammatory markers in acute coronary syndrome. Bmc Cardiovascular Disorders 2021; 21. Article. DOI: 10.1186/s12872-021-02236-7.
2. Lamichhane P, Agrawal A, Abouainain Y, et al. Utility of neutrophil-to-high-density lipoprotein-cholesterol ratio in patients with coronary artery disease: a narrative review. Journal of International Medical Research 2023; 51. Review. DOI: 10.1177/03000605231166518.
3. Soehnlein O. Multiple Roles for Neutrophils in Atherosclerosis. Circulation Research 2012; 110: 875-888. Review. DOI: 10.1161/circresaha.111.257535.
4. Sager HB and Nahrendorf M. Inflammation: a trigger for acute coronary syndrome. Quarterly Journal of Nuclear Medicine and Molecular Imaging 2016; 60: 185-193. Review.
5. Tian L, Li C, Liu Y, et al. The Value and Distribution of High-Density Lipoprotein Subclass in Patients with Acute Coronary Syndrome. Plos One 2014; 9. Article. DOI: 10.1371/journal.pone.0085114.
6. Guo J, Chen M, Hong Y, et al. Comparison of the Predicting Value of Neutrophil to high-Density Lipoprotein Cholesterol Ratio and Monocyte to high-Density Lipoprotein Cholesterol Ratio for in-Hospital Prognosis and Severe Coronary Artery Stenosis in Patients with ST-Segment Elevation Acute Myocardial Infarction Following Percutaneous Coronary Intervention: A Retrospective Study. Journal of Inflammation Research 2023; 16: 4541-4557. Article. DOI: 10.2147/jir.S425663.
7. Chen Y, Jiang D, Tao H, et al. Neutrophils to high-density lipoprotein cholesterol ratio as a new prognostic marker in patients with ST-segment elevation myocardial infarction undergoing primary percutaneous coronary intervention: a retrospective study. Bmc Cardiovascular Disorders 2022; 22. Article. DOI: 10.1186/s12872-022-02870-9.
8. Huang J-B, Chen Y-S, Ji H-Y, et al. Neutrophil to high-density lipoprotein ratio has a superior prognostic value in elderly patients with acute myocardial infarction: a comparison study. Lipids in Health and Disease 2020; 19. Article. DOI: 10.1186/s12944-020-01238-2.
9. Ozgeyik M and Ozgeyik MO. LONG-TERM PROGNOSIS AFTER TREATMENT OF TOTAL OCCLUDED CORONARY ARTERY IS WELL PREDICTED BY NEUTROPHIL TO HIGH-DENSITY LIPOPROTEIN RATIO: A COMPARISON STUDY. Kardiologiya 2021; 61: 60-67. Article. DOI: 10.18087/cardio.2021.7.n1637.
10. Kou T, Luo H and Yin L. Relationship between neutrophils to HDL-C ratio and severity of coronary stenosis. Bmc Cardiovascular Disorders 2021; 21. Article. DOI: 10.1186/s12872-020-01771-z.
11. Gao J, Lu J, Sha W, et al. Relationship between the neutrophil to high-density lipoprotein cholesterol ratio and severity of coronary artery disease in patients with stable coronary artery disease. Frontiers in Cardiovascular Medicine 2022; 9. Article. DOI: 10.3389/fcvm.2022.1015398.
12. Ren H, Zhu B, Zhao Z, et al. Neutrophil to high-density lipoprotein cholesterol ratio as the risk mark in patients with type 2 diabetes combined with acute coronary syndrome: a cross-sectional study. Scientific Reports 2023; 13. Article. DOI: 10.1038/s41598-023-35050-6.
13. Li J, Liu M, Liu F, et al. Age and Genetic Risk Score and Rates of Blood Lipid Changes in China. Jama Network Open 2023; 6. Article. DOI: 10.1001/jamanetworkopen.2023.5565.
14. Cheung BMY, Li M, Ong KL, et al. High density lipoprotein-cholesterol levels increase

with age in American women but not in Hong Kong Chinese women. Clinical Endocrinology 2009; 70: 561-568. Article. DOI: 10.1111/j.1365-2265.2008.03361.x.

15. Xu W, Guan H, Gao D, et al. Sex-specific association of monocyte count to high-density lipoprotein ratio with SYNTAX score in patients with suspected stable coronary artery disease. Medicine 2019; 98. Article. DOI: 10.1097/md.0000000000017536.

16. Manoochehri H, Gheitasi R, Pourjafar M, et al. Investigating the relationship between the severity of coronary artery disease and inflammatory factors of MHR, PHR, NHR, and IL-25. Medical journal of the Islamic Republic of Iran 2021; 35: 85-85. DOI: 10.47176/mjiri.35.85.

17. Chen Y, Zhang Y, Zhao G, et al. Difference in Leukocyte Composition between Women before and after Menopausal Age, and Distinct Sexual Dimorphism. Plos One 2016; 11. Article. DOI: 10.1371/journal.pone.0162953.

18. Wang J, Huang X, Fu C, et al. Association between triglyceride glucose index, coronary artery calcification and multivessel coronary disease in Chinese patients with acute coronary syndrome. Cardiovascular Diabetology 2022; 21. Article. DOI: 10.1186/s12933-022-01615-4.

19. Alberti K, Zimmet PZ and Consultation WHO. Definition, diagnosis and classification of diabetes mellitus and its complications part 1: Diagnosis and classification of diabetes mellitus - Provisional report of a WHO consultation. Diabetic Medicine 1998; 15: 539-553. Article. DOI: 10.1002/(sici)1096-9136(199807)15:7<539::Aid-dia668>3.0.Co;2-s.

20. Chen J, Chen M-H, Li S, et al. Usefulness of the Neutrophil-to-Lymphocyte Ratio in Predicting the Severity of Coronary Artery Disease: A Gensini Score Assessment. Journal of Atherosclerosis and Thrombosis 2014; 21: 1271-1282. Article. DOI: 10.5551/jat.25940.

21. Gensini GG. A MORE MEANINGFUL SCORING SYSTEM FOR DETERMINING THE SEVERITY OF CORONARY HEART-DISEASE. American Journal of Cardiology 1983; 51: 606-606. Letter. DOI: 10.1016/s0002-9149(83)80105-2.

22. Hiles M, Simmons A, Hilleman D, et al. Atherosclerotic Cardiovascular Disease in Women: Providing Protection With Lipid-altering Agents. Clinical Therapeutics 2023; 45: 1127-1136. Review. DOI: 10.1016/j.clinthera.2023.08.021.

23. Harslof M, Pedersen KM, Nordestgaard BG, et al. Low High-Density Lipoprotein Cholesterol and High White Blood Cell Counts A Mendelian Randomization Study. Arteriosclerosis Thrombosis and Vascular Biology 2021; 41: 976-987. Article. DOI: 10.1161/atvbaha.120.314983.

24. Liu M, Liu X and Wei Z. MHR and NHR but not LHR were associated with coronary artery disease in patients with chest pain with controlled LDL-C. J Investig Med 2022; 70: 1501-1507. Article. DOI: 10.1136/jim-2021-002314.

25. Calabrò P, Niccoli G, Gragnano F, et al. Are we ready for a gender-specific approach in interventional cardiology? Int J Cardiol. 2019 Jul 1;286:226-233. DOI: 10.1016/j.ijcard.2018.11.022.

26. Newson L. Menopause and cardiovascular disease. Post reproductive health 2018; 24: 44-49. ; Review. DOI: 10.1177/2053369117749675.

27. Mendelsohn ME and Karas RH. The protective effects of estrogen on the cardiovascular system. New England Journal of Medicine 1999; 340: 1801-1811. Review. DOI: 10.1056/nejm199906103402306.

28. Hodis HN, Mendelsohn ME, Langer RD, et al. Protective effects of estrogen on the cardiovascular system - Discussion. American Journal of Cardiology 2002; 89: 17E-18E.

Editorial Material.
29. Xiang D, Liu Y, Zhou S, et al. Protective Effects of Estrogen on Cardiovascular Disease Mediated by Oxidative Stress. Oxidative Medicine and Cellular Longevity 2021; 2021. Review. DOI: 10.1155/2021/5523516.
30. Molloy EJ, O'Neill AJ, Grantham JJ, et al. Sex-specific alterations in neutrophil apoptosis: the role of estradiol and progesterone. Blood 2003; 102: 2653-2659. Article. DOI: 10.1182/blood-2003-02-0649.
31. Azene ZN, Davis SR, McNeil JJ, et al. Estrone, sex hormone binding globulin and lipid profiles in older women: an observational study. Climacteric 2023; 26: 114-120. Article. DOI: 10.1080/13697137.2023.2165908.
32. Trtica Majnaric L, Guljas S, Bosnic Z, et al. Neutrophil-to-Lymphocyte Ratio as a Cardiovascular Risk Marker May Be Less Efficient in Women Than in Men. Biomolecules 2021; 11. Review. DOI: 10.3390/biom11040528.
33. Otsuka K, Ishikawa H, Shimada K, et al. Low-Attenuation Coronary Plaque Volume and Cardiovascular Events in Patients with Distinct Metabolic Phenotypes with or without Diabetes. Rev Cardiovasc Med. 2023 Dec 25;24(12):361. DOI: 10.31083/j.rcm2412361.
34. Pan Q, Zhang W, Li X, et al. Sex Difference in the Association Between Neutrophil to Lymphocyte Ratio and Severity of Coronary Artery Disease. Angiology 2022; 73: 470-477. Article. DOI: 10.1177/00033197211070884.
35. Luo H, Kou T and Yin L. High-Sensitivity C-Reactive Protein to HDL-C Ratio A Predictor of Coronary Artery Disease. International Heart Journal 2021; 62: 1221-1229. Article. DOI: 10.1536/ihj.21-246.
36. Yuan S, Li L, Pu T, et al. The relationship between NLR, LDL-C/HDL-C, NHR and coronary artery disease. PLoS One. 2024;19(7):e0290805. DOI:10.1371/journal.pone.0290805.
